# Operationalising a Comprehensive Lactation Management Centre in a Tertiary NICU in North India: A Retrospective Implementation Study With Before-After Comparison of Breastfeeding and Counselling Coverage

**DOI:** 10.7759/cureus.118825

**Published:** 2026-10-30

**Authors:** Anu Sharma, Aishwarya Kashyap, Vaishnavi Pandey, Alok Kumar Singh, Muskan Sharma, Ashok Kumar

**Affiliations:** 1 Pediatrics/Neonatology, Institute of Medical Sciences, Banaras Hindu University, Varanasi, IND; 2 Pediatrics, Institute of Medical Sciences, Banaras Hindu University, Varanasi, IND; 3 Public Health Research, Community Empowerment Lab (CEL), Varanasi, IND; 4 Pediatrics and Child Health, Institute of Medical Sciences, Banaras Hindu University, Varanasi, IND; 5 Pediatrics, Heritage Institute of Medical Sciences, Varanasi, IND

**Keywords:** comprehensive lactation management centre, exclusive breastfeeding, mothers own milk, neonatal intensive care unit, pasteurised donor human milk

## Abstract

Background: Feeding practices in neonatal intensive care units (NICUs) are often compromised by limited lactation support and mother-infant separation. A Comprehensive Lactation Management Centre (CLMC) integrates counselling, bedside lactation assistance and donor human milk banking within the NICU. Evidence on the real-world operationalisation of such centres in tertiary care institutions in India remains limited. This study describes the operationalisation of a CLMC in a tertiary NICU and evaluates its effect on exclusive breastfeeding and structured counselling coverage through a before-after comparison.

Methodology: This retrospective implementation study was conducted in the NICU and CLMC of a public-sector tertiary centre with 4,200-4,500 deliveries annually. Baseline feeding and counselling practices (April-June 2024) were compared with those in the post-implementation period (July 2024-July 2025). Core components included structured lactation counselling, bedside milk expression support, a functional donor human milk pathway, daily multidisciplinary rounds and WhatsApp-based coordination. Feeding and counselling indicators during the baseline and post-implementation periods, monthly operational trends and donor profiles were analysed.

Results: The proportion of mothers receiving structured breastfeeding counselling rose from 87/353 (25%) to 1,613/1,841 (88%). Exclusive breastfeeding at discharge increased from 25/56 (45%) to 436/534 (82%). Over the implementation period, 541 neonates received pasteurised donor human milk (PDHM), and the peak monthly donation volume reached 30,009 ml. Early initiation of milk expression, together with hands-on demonstration and lactation counselling, appeared to drive the change. Mother-infant separation and perceived low milk supply remained persistent barriers.

Conclusions: CLMC establishment was associated with improved exclusive breastfeeding and structured breastfeeding counselling coverage. As a retrospective analysis, these findings reflect associations rather than causal effects. Sustaining these gains requires ongoing lactation support and system-level coordination.

## Introduction

Human milk is the ideal nutrition and recommended first feed for all newborns, including sick and preterm neonates [1]. In low- and middle-income countries (LMICs), exclusive breastfeeding and early human milk feeding in the NICU remain suboptimal [2], even though the evidence for lower rates of necrotising enterocolitis (NEC) and late-onset sepsis and for better neurodevelopmental outcomes is strong [3]. When mother's own milk (MOM) is unavailable or insufficient, pasteurised donor human milk (PDHM) is the next best option for vulnerable neonates [4].

In India, the National Guidelines on Lactation Management Centres in Public Health Facilities (MoHFW 2017) recommend setting up Comprehensive Lactation Management Centres (CLMCs) in tertiary facilities, bringing breastfeeding promotion, milk expression support and donor human milk banking together under one service [5]. A CLMC differs from a stand-alone donor milk bank because it embeds counselling, bedside lactation care, milk expression support and donor milk services within the routine NICU workflow.

Despite this policy endorsement, implementation in high-volume public-sector tertiary centres is often uneven. Common obstacles are too few dedicated lactation staff, competing clinical demands in overcrowded NICUs, gaps in infrastructure, and the need for continued behavioural support for mothers coping with separation from their infants [6].

Published implementation experiences from North Indian public-sector tertiary NICUs remain scarce, and these settings differ substantially from urban private units in delivery volumes and resource constraints.

We report the operationalisation of a CLMC in our NICU between July 2024 and July 2025. Our objectives were to (1) describe how the CLMC was set up and adapted, using prospectively maintained operational records; (2) evaluate its effect on breastfeeding and counselling coverage through a retrospective before-after comparison; and (3) analyse trends in donor human milk collection and utilisation, the microbiological quality of donor milk, and donor mothers’ profiles.

## Materials and methods

Study design and setting

This was a retrospective implementation study with a before-after comparison of feeding outcomes. It was conducted in the 40-bedded Level-III NICU of a public-sector tertiary teaching hospital that serves eastern Uttar Pradesh and the adjoining districts of Bihar. The institution records 4,200-4,500 deliveries annually, with a high proportion of preterm, low-birth-weight and referred-in sick neonates. Ethical approval was obtained from the Institutional Ethics Committee, Institute of Medical Sciences, Banaras Hindu University (IMS-BHU) (Ref: IMS/IEC/FEBRUARY/2026/319, 12 March 2026).

The baseline period was April-June 2024. Structured counselling coverage was derived from mothers whose babies required NICU admissions (353 at baseline and 1,841 during implementation). Exclusive breastfeeding at NICU discharge predominantly included neonates who were sicker in the initial days and required growth monitoring and supportive supervision in the mother-newborn care unit before discharge (56 neonates at baseline and 534 during implementation). The implementation period was July 2024-July 2025. We defined structured breastfeeding counselling as at least one counselling session with a hands-on demonstration of milk expression [7]. Before CLMC operationalisation, structured breastfeeding counselling was not routinely offered to all mothers, and the counselling provided followed a passive rather than proactive approach. Exclusive breastmilk feeding at NICU discharge was defined as the neonate receiving only breastfeeding and/or MOM in the 24 hours before discharge [8,9].

Description of the Intervention

The CLMC comprised the following components.

Provision of training to staff working in the NICU, postnatal wards, labour rooms and operation theatres. This included small, focused discussions, knowledge-sharing sessions and practical skills training using breast models. Correct latching, infant positioning, manual milk expression, operation of hospital-grade breast pumps, and recognition and management of common breastfeeding problems, including breast engorgement, mastitis and inverted nipples, were also taught.

Structured delivery-room and postnatal breastfeeding counselling for mothers of neonates admitted to the NICU. Bedside and dedicated-room milk expression support, with emphasis on demonstrating hand expression alongside mechanical expression where available. A functional donor human milk pathway, including donor screening and serology (full criteria in Table 1), hygienic milk collection, Holder pasteurisation, microbiological safety checks, cold-chain storage and prescription-based issue of PDHM to eligible neonates when MOM was insufficient or unavailable.

**Table 1:** Donor screening criteria followed in our Comprehensive Lactational Management Centre. HIV, human immunodeficiency virus; TB, tuberculosis; HBV, hepatitis B virus; HCV, hepatitis C virus

Donor screening criteria
Prerequisite: A lactating woman who was in good health, fed her baby satisfactorily, was willing to undergo blood testing if required, and was enthusiastic about becoming a breast milk donor.
Examination and serology:
1. Physical examination of the breast to check for the presence of skin or breast lesions was performed.
2. Serological reports available for, HIV 1 or 2, Hepatitis B, Syphilis/VDRL and Hepatitis C. If not available in the past six months, repeat testing required
Milk for donation was not taken if:
Blood transfusion/organ or tissue transplant within the past 12 months
Permanent tattoo/body piercing using instruments other than single-use instruments within the last 12 months
Daily use of alcohol
Use of tobacco/nicotine products, including gum or e-cigarettes
Chronic Infection like HIV, active TB, Hep B, Hep C, etc., or a history of cancer within the last three years.
A sexual partner in the past 12 months who is suffering from/at risk for HBV, HCV (optional), HIV and venereal diseases or one who has high risk behaviour for contracting them

Daily multidisciplinary NICU rounds incorporating feeding-plan review for every admitted neonate, with explicit assessment of MOM availability, volume and mode of delivery. A WhatsApp-based communication channel among clinicians, nursing staff and the CLMC manager enabled real-time problem-solving around donor availability, mother-side barriers, feeding challenges and milk-handling processes, especially during evening and night hours. Structured behaviour-change support addressed perceived low milk supply, sustaining milk expression during prolonged separation of mother and infant, and integrating kangaroo mother care (KMC) to support lactation. The team included a paediatrician, a neonatologist, neonatology fellows, junior residents, nursing officers, a CLMC manager, a laboratory technician, hygiene support staff, and a microbiology team comprising a microbiologist and a laboratory technician. One dedicated lactation counsellor and one staff nurse were available during the daytime in each shift. At night, only one staff nurse was available, serving as both counsellor and nurse. The implementation was supported by a system-strengthening facilitatory programme (LIFT-UP), which provided comprehensive training, supportive supervision and mentorship for CLMC-related activities in the unit.

CLMC operational pathway is presented in Figure 1.

**Figure 1:**
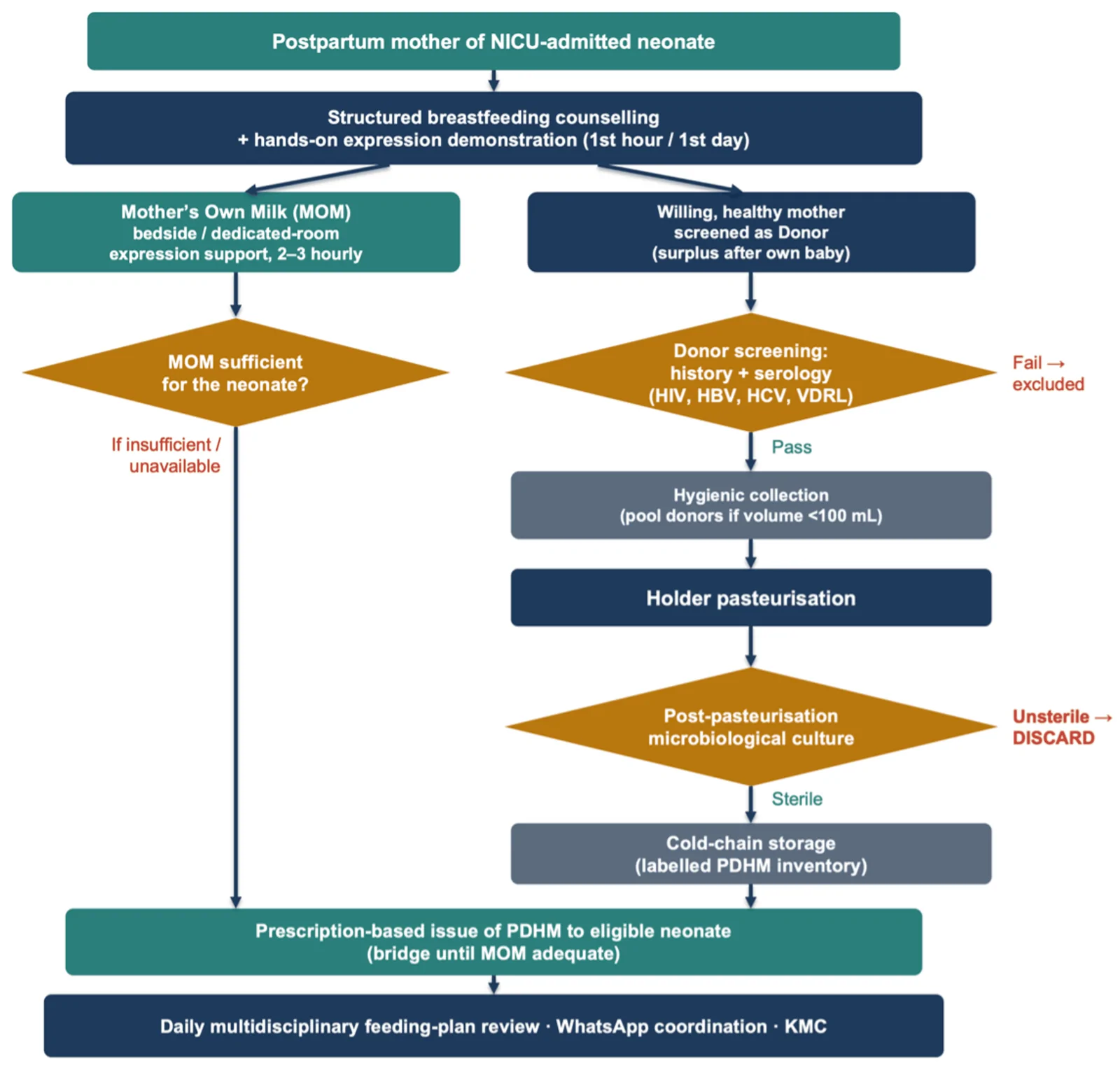
Operational pathway for Comprehensive Lactational Management Centre (CLMC). Original schematic illustration developed by the study team to represent the operational workflow of the CLMC (Draw.io, JGraph Ltd., Northampton, UK). NICU, neonatal intensive care unit; HIV, human immunodeficiency virus; HBV, hepatitis B virus; HCV, hepatitis C virus; VDRL, Venereal Disease Research Laboratory; PDHM, pasteurised donor human milk; KMC, kangaroo mother care.

Operational Review Mechanism

Implementation was monitored through weekly/monthly review meetings involving the NICU team and CLMC staff. These meetings examined service volumes, donor availability, mother-side bottlenecks and operational challenges. Adaptations such as revised counselling content, workflow adjustments for night-shift expression support and measures to address milk contamination rates were made iteratively in response to these reviews. No formal quality-improvement methodology was applied.

Data Collection

For the baseline period, data were retrospectively abstracted from NICU admission registers and discharge records before CLMC operationalisation. For the post-implementation period, data were drawn from prospectively maintained CLMC service registers, postnatal round registers and feeding records. All data were de-identified before analysis. EBF and counselling analyses included mothers and babies with documented details. Donor analysis excluded 29 donors with missing data on maternal age, parity, gestational age at delivery, maternal risk factors and need for NICU admission.

Primary outcomes were (1) the proportion of mothers receiving structured breastfeeding counselling and (2) the proportion of neonates exclusively breastfed at NICU discharge. Secondary indicators were the number of donors, the number of neonates receiving PDHM, the peak monthly donation volume, the microbiological status of PDHM and donor mothers’ profiles.

Before-After Comparison

EBF and structured breastfeeding counselling proportions from the baseline period were compared with those from the post-implementation period. Because this was a retrospective implementation design, the comparison is descriptive and intended to show the direction and magnitude of change associated with CLMC operationalisation, not to establish a causal effect. 

Statistical Analysis

EBF and structured breastfeeding counselling proportions were compared descriptively using absolute differences between the baseline and post-implementation periods. Inferential testing for before-after feeding comparisons was not applied because differences in data quality between the baseline and post-implementation periods precluded valid causal inference; the RRs quantify magnitude only. Interrupted time-series regression was fitted to the 16 monthly data points (three baseline and 13 implementation), estimating the pre-intervention level and slope, the immediate level change at CLMC onset, and the post-intervention change in slope. The within-implementation trend was also examined using a binomial segmented regression model. The monthly post-pasteurisation contamination rate was plotted on a statistical process control (p-) chart with 3-sigma control limits.

For donor-characteristic comparisons, categorical variables are presented as numbers (%) and continuous variables as mean ± SD for normally distributed data or median (IQR) for non-normally distributed data. Between-group comparisons used the chi-square or Fisher’s exact test for categorical variables and the independent-samples t-test for normally distributed continuous variables. All tests were two-tailed, with statistical significance set at *P* < 0.05. Multivariable binary logistic regression was applied to assess factors associated with donation volumes above and below the sample median (above 185 mL versus 185 mL or below), adjusting for maternal age, risk factors, mode of delivery, NICU admission and gestational age. Monthly operational trends are described using tabular data (absolute counts and volumes) and graphs. Donor-characteristic comparisons and the multivariable logistic regression of donation volume were performed in JASP version 0.96.0 (JASP Team). The remaining analyses were performed in Stata version 19.5 (StataCorp, College Station, TX). The Standards for Reporting Implementation Studies (StaRI) and the Template for Intervention Description and Replication (TIDieR) are reported in Appendices A-B.

## Results

The initial operating model, implementation experience, challenges and adaptations were derived from prospectively maintained CLMC service registers, weekly/monthly review meetings, postnatal round registers, the WhatsApp group archive and the event log initiated in December 2024. No formal qualitative methods (e.g., structured interviews, focus groups or thematic analysis) were used; the narrative reflects only the documented operational records.

Establishment and initial operating model

The CLMC began functioning in July 2024 in the dedicated CLMC area within the existing NICU. As per the national guidelines, our centre had designated areas for counselling, handwashing, milk expression, pasteurisation, storage, thawing and dispensing, equipment cleaning and sterilisation, and record maintenance. A laminar flow system maintained sterility during sample pooling and microbiological culture sampling. According to the CLMC service register, early work focused on setting aside physical space for milk expression, establishing donor screening protocols in accordance with the 2017 National Guidelines (Table 1) [10,11], assigning dedicated counselling staff, and incorporating CLMC-specific items into the daily NICU and postnatal ward rounds. According to the milk-bank log, when a single donor’s milk volume was below 100 mL, milk from more than one donor was pooled to reach the minimum sample volume. The Holder method of pasteurisation was used. The cold chain was strictly maintained at −20 °C for pre- and post-pasteurisation samples and during transport. Microbial testing was performed only after pasteurisation, and a sample from each batch of PDHM was sent to the microbiology laboratory to confirm sterility before dispensing. Samples reported as unsterile were discarded, as documented in the milk-bank log. Review records show that the initial model emphasised process strengthening through structured counselling, routine bedside expression support, and explicit feeding-plan documentation for every admitted neonate.

Implementation experience

Review records from the first two to three months noted that starting lactation counselling and expression support within the first hour and the first day of admission was associated with higher documented milk output in the CLMC service register. Structured 2-3 hourly expression cycles, reinforced by bedside demonstration by staff nurses, were recorded as the standard operating procedure from the first month onward. Hand expression was the main method used for mothers of very preterm or sick neonates in the early postpartum days. The CLMC functioned as a referral point where clinicians, staff nurses and the CLMC manager worked together to address individual mother-side barriers to expression and breastfeeding, rather than as a stand-alone milk-collection facility.

Challenges and adaptations

Separation of mother and infant because of NICU admission was the most frequently documented barrier to early and sustained lactation, despite gains in early initiation. Perceived low milk supply was the maternal concern most often recorded in counsellor notes. Postnatal round registers showed that donor recruitment required sustained counsellor-led outreach in the postnatal wards and did not become self-sustaining during the study period. Heavy microbial contamination and errors in handling PDHM were additional challenges documented.

Adaptations during the implementation period included WhatsApp-based coordination for after-hours queries, revised counselling content that directly addressed perceived insufficient milk, and stronger night-shift expression support. The event log records that responsibility for microbiology-related processes was handed over to the CLMC technician in October 2024. This followed microbial growth detected in post-pasteurisation samples in September (20/47, 42.6%, of batches were unsterile) and October 2024 (13/46, 28.3%). As the pasteuriser temperature had been consistently well maintained, the heavy contamination was attributed instead to inadequate cleaning of the valve diaphragm in the breast-pump suction kit, and the hygiene staff were subsequently retrained. From December 2024, a structured event log was established to document, audit and correct data-entry discrepancies in CLMC records and errors in handling PDHM, particularly at night. Problems with CLMC equipment and its functional status were also recorded in this log.

Quantitative outcomes

The proportion of mothers receiving structured breastfeeding counselling rose from 87/353 (25%) at baseline to 1,613/1,841 (88%) after implementation. Beyond coverage, counselling was delivered at a high dose, with 2,155 CLMC counselling contacts recorded over the period and a further 6,864 in-ward counselling sessions documented from October 2024. Exclusive breastfeeding at NICU discharge increased from 25/56 (45%) to 436/534 (82%) (Table 2).

**Table 2:** Feeding and service outcomes before and after CLMC operationalisation. Baseline period: April-June 2024. Implementation period: July 2024-July 2025. Denominators: Structured counselling is expressed per mother whose baby/babies required NICU admission (baseline *N *= 353; post-implementation *N *= 1,841). Exclusive breastfeeding is expressed among neonates with a documented discharge feeding mode (baseline *N* = 56; post-implementation *N* = 534), as recorded in the neonatal feeding register. Relative risks quantify magnitude only. The study design does not permit causal inference because of its retrospective nature and data asymmetry. Structured breastfeeding counselling was defined as at least one counselling session coupled with a hands-on demonstration of milk expression [7]. Exclusive breastmilk feeding at NICU discharge was defined as the neonate receiving only breastfeeding and/or MOM in the 24 hours before discharge, consistent with the Vermont Oxford Network (VON) operational discharge-feeding criteria and the WHO definition of exclusive breastfeeding [8,9]. CLMC, Comprehensive Lactation Management Centre; PDHM, pasteurised donor human milk

Outcome indicator	Baseline (Apr-Jun 2024), *n*/*N* (%)	Post-implementation (Jul 2024-Jul 2025), *n*/*N* (%)	Absolute change	Relative risk (RR)
Mothers receiving structured breastfeeding counselling (%)	87/353 (25%)	1,613/1,841 (88%)	+63	3.53
Exclusive breastmilk feeding at NICU discharge (%)	25/56 (44.6%)	436/534 (81.6%)	+37	1.83

Interrupted time-series analysis suggested an immediate increase in exclusive breastfeeding of approximately 36 percentage points at CLMC implementation (95% CI 24.6-47.2; *P* < 0.001), with binomial segmented regression yielding an OR of 5.38 (95% CI 1.61-17.9; quasi-binomial dispersion 0.60). However, the availability of only three pre-implementation months limited reliable estimation of the baseline trend, making the estimated change dependent on baseline extrapolation (Figure 2).

**Figure 2:**
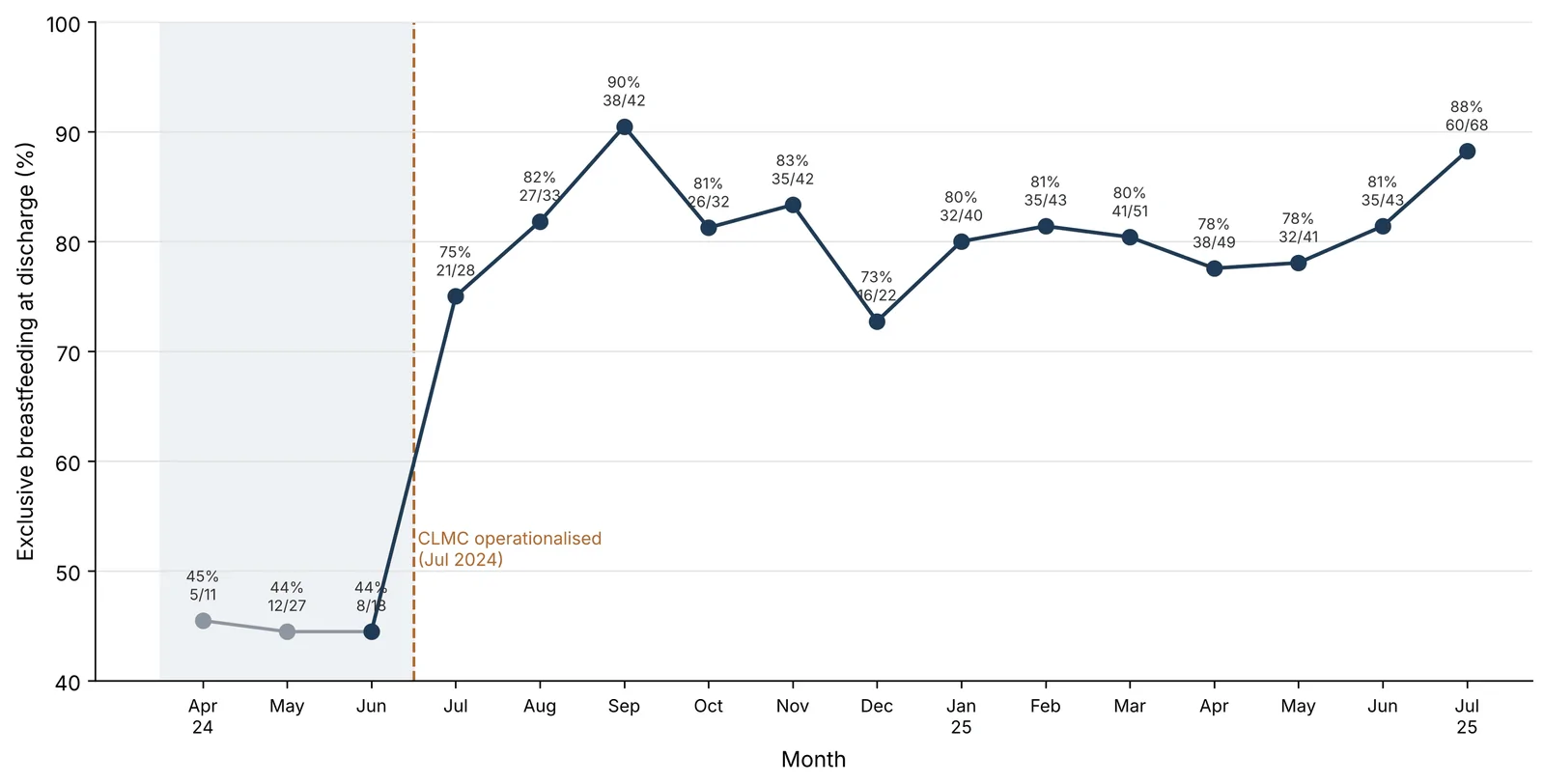
Monthly exclusive breastfeeding at discharge during CLMC implementation (July 2024-July 2025), with the baseline period (April-June 2024) and fitted trend line shown. CLMC, Comprehensive Lactation Management Centre

Of the 241 donor mothers, descriptive analysis was performed for 212 (88.0%), comprising 117 term (55.2%) and 95 preterm (44.8%) mothers; the remaining 29 (12.0%) were excluded because of incomplete study records (Figure 3).** **

**Figure 3:**
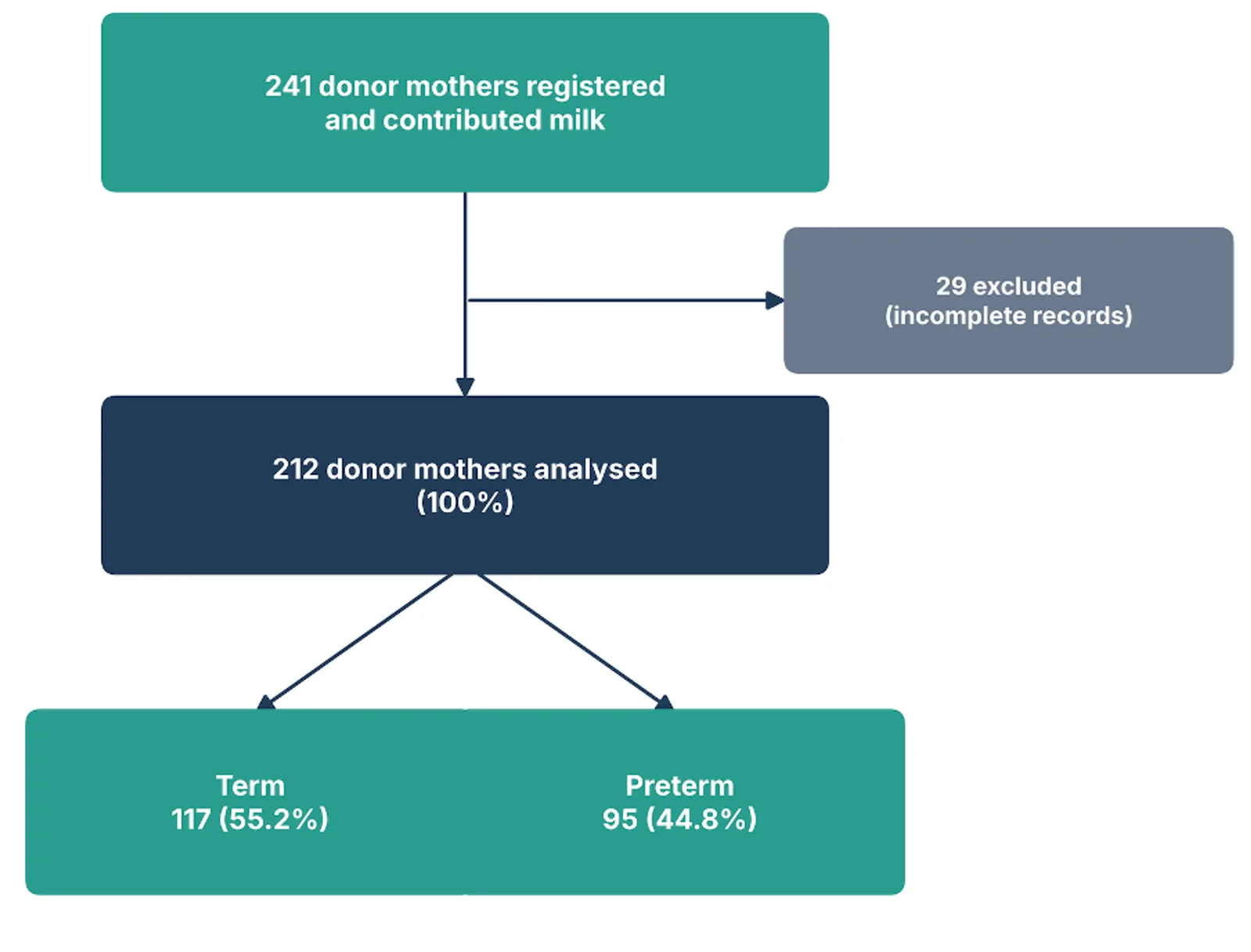
Donor flow diagram showing the number of donors and recipients.

Of the 241 donor mothers, descriptive analysis was performed for 212 (88.0%; 117 term and 95 preterm); the remaining 29 (12.0%) were excluded because of incomplete study records (Figure 3). Mean maternal age was 27.06 ± 3.39 years in the term group and 27.26 ± 4.42 years in the preterm group. Most donors had a lower-segment caesarean delivery, and 76/95 (80%) of mothers who delivered prematurely had health problems. Maternal age, parity, occupation and residence were comparable between groups. NICU admission and maternal risk factors were significantly more common among preterm donor mothers. Skin-to-skin contact (term: 80/117, 68.4%, vs. preterm: 25/95, 26.3%; *P* < 0.001) and early initiation of breastfeeding (term: 66/117, 56.4%, vs. preterm: 18/95, 18.9%; *P* < 0.001) were significantly less common among preterm donor mothers. Mothers of preterm infants contributed a larger share of the total milk volume (92,952/133,542 mL, 69.6%) than mothers of term infants (40,590/133,542 mL, 30.4%), although per-donor volume did not differ significantly between groups (overall median 185 mL, IQR 83.75-391.25; *P* = 0.184) (Table 3).

**Table 3:** Baseline characteristics of donor mothers (term vs. preterm). ^a^Chi-square test. ^b^Fisher's exact test. ^c^Mann-Whitney test. Effect size is reported as phi (φ) for 2 × 2 tables and Cramér's V for larger tables. Continuous age was compared using Welch's t-test (Cohen's d). For sex of the baby, Pearson's χ² test without continuity correction is reported (Yates-corrected χ² = 0.00, *P* = 1.00). Donation volume is reported as median (IQR) and compared using the Mann-Whitney U test (rank-biserial *r*). The term/preterm split (40,590 + 92,952 = 133,542 mL) covers the 212 analysed donors; the difference of approximately 6,546 mL from the overall total of 140,088 mL represents the volume contributed by the 29 donors excluded from the descriptive analysis. df, degrees of freedom; EIBF, early initiation of breastfeeding; IQR, interquartile range; LSCS, lower segment caesarean section; NICU, neonatal intensive care unit; SD, standard deviation; SSC, skin-to-skin contact

Variable	Term donors (*n* = 117)	Preterm donors (*n* = 95)	Test statistic (df)	P	Effect size
Age, mean ± SD (years)	27.06 ± 3.39	27.26 ± 4.42	*t* = 0.37 (Welch)	0.713	*d* = 0.05
Age band (years)			χ² = 2.94 (2)^a^	0.230	*V* = 0.12
≤20	2 (1.7%)	4 (4.2%)			
21-30	99 (84.6%)	72 (75.8%)			
≥31	16 (13.7%)	19 (20.0%)			
Total	117 (100%)	95 (100%)			
Parity			Fisher exact^b^	1.000	φ = 0.01
Primipara	60 (51.3%)	48 (50.5%)			
Multipara	57 (48.7%)	47 (49.5%)			
Mode of delivery			χ² = 0.61^a^	0.436	φ = 0.05
LSCS	91 (77.8%)	78 (82.1%)			
Vaginal delivery	26 (22.2%)	17 (17.9%)			
Occupation			χ² = 0.89^a^	0.346	φ = 0.07
Employed	16 (13.7%)	9 (9.5%)			
Housewife	101 (86.3%)	86 (90.5%)			
Residence			χ² = 0.03^a^	0.856	φ = 0.01
Urban	47 (40.2%)	37 (38.9%)			
Rural	70 (59.8%)	58 (61.1%)			
NICU admission of baby (yes)	49 (41.9%)	83 (87.4%)	χ² = 46.17^a^	<0.001	φ = 0.47
Maternal risk factors present	17 (14.5%)	76 (80.0%)	χ² = 91.26 ^a^	<0.001	φ = 0.66
Sex of baby (*n* = 225, incl. 13 sets of twins; 2 term and 11 preterm)	*n* = 119	*n* = 106	χ² = 0.01 (1)^a^	0.911	φ = 0.01
Male	66 (55.5%)	58 (54.7%)	χ² = 0.01 (1)^a^	0.911	φ = 0.01
Female	53 (44.5%)	48 (45.3%)	χ² = 0.01 (1)^a^	0.911	φ = 0.01
Skin-to-skin contact (SSC) - yes	80 (68.4%)	25 (26.3%)	χ² = 37.10 (1)	<0.001	φ = 0.42
Early initiation of breastfeeding (EIBF)- yes	66 (56.4%)	18 (18.9%)	χ² = 30.76 (1)	<0.001	φ = 0.38
Volume per donor, median mL (IQR)	135 (80-325)	205 (110-452.5)	*U* = 4704 (*z* = 1.92)^c^	0.055	*r* = 0.15
Total volume donated, mL (% of total)	40,590 (30.4%)	92,952 (69.6%)			
Median volume per donor (overall), mL (IQR)	185 (83.75-391.25)				

Although mothers of NICU-admitted neonates donated more milk in the univariable analysis (median 218 vs. 128 mL; Mann-Whitney *P* = 0.004), this association did not persist after adjustment (aOR 1.61; *P* = 0.186). This indicates that the crude association was not independent and that the sample may have been underpowered for multivariable modelling of donor milk volume (Table 4).

**Table 4:** Multivariable logistic regression analysis of factors associated with above-median donation volume (>185 mL vs. ≤185 mL). Variance inflation factors (VIFs) were all <2.5, indicating no problematic collinearity. The overall model was not statistically significant (likelihood-ratio χ², *P* = 0.235; pseudo-*R*² = 0.031), and no factor was independently associated with above-median donation volume. SSC, skin-to-skin contact; NICU, neonatal intensive care unit; EIBF, early initiation of breastfeeding; aOR, adjusted odds ratio

Predictor	aOR	95% CI	P	VIF
Maternal age (per year)	1.02	0.95-1.10	0.604	1.02
Term gestation (vs. preterm)	0.73	0.33-1.63	0.445	2.08
NICU admission (yes vs. no)	1.61	0.79-3.29	0.186	1.60
SSC (yes vs. no)	0.70	0.30-1.65	0.416	2.43
EIBF (yes vs. no)	1.17	0.51-2.69	0.712	2.14
Vaginal delivery (vs. caesarean)	1.48	0.74-2.97	0.270	1.02
Maternal risk factors (no vs. yes)	0.86	0.41-1.81	0.695	1.78

Monthly donation volume peaked at 30,009 mL. This sudden rise was driven largely by a few mothers who donated substantially more than the average donor, although it also reflected the maturation of donor recruitment, pasteurisation throughput and clinical uptake of PDHM in July 2025 (Figure 4). 

**Figure 4:**
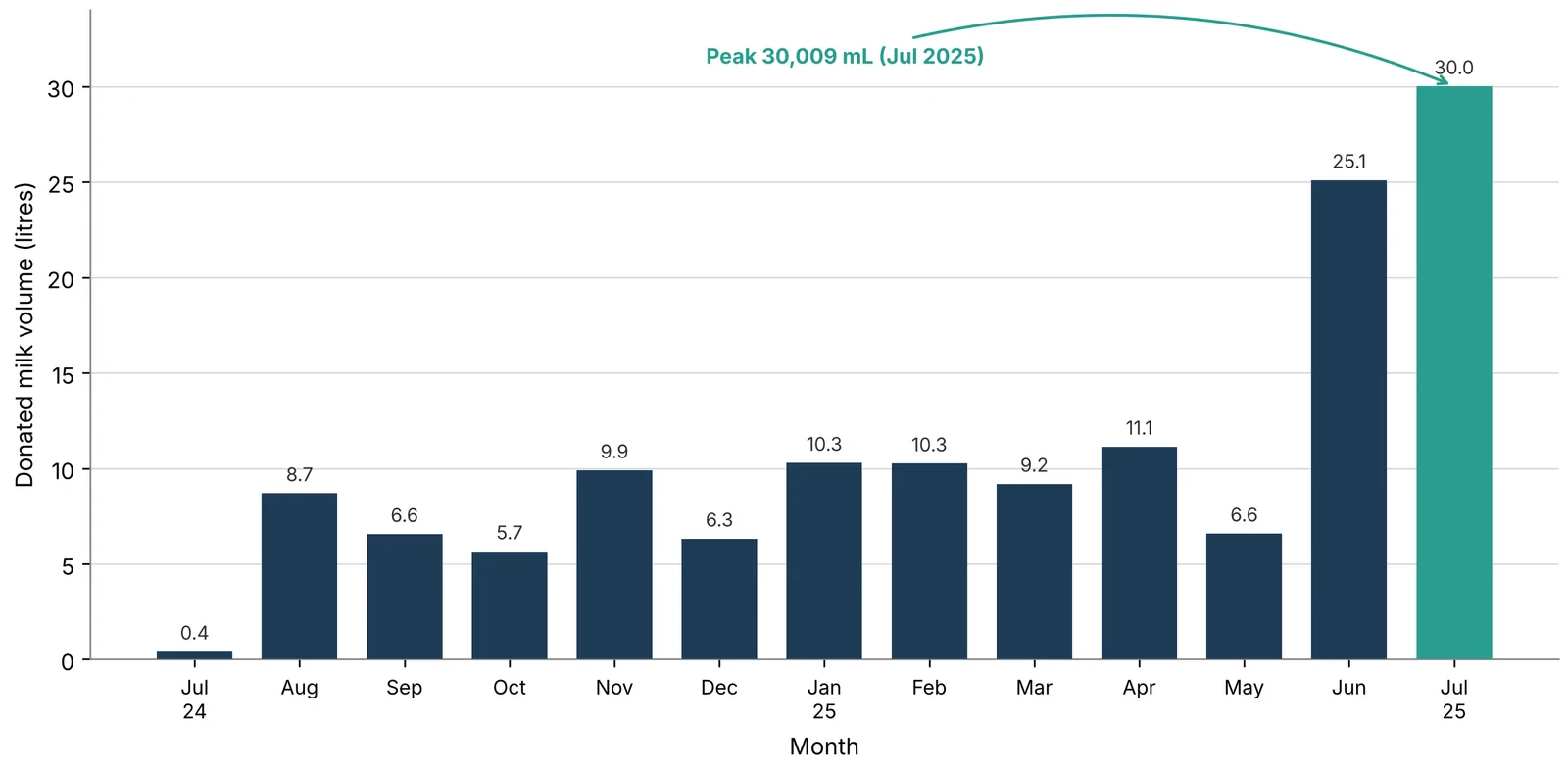
Monthly milk donation trends in the CLMC. CLMC, Comprehensive Lactation Management Centre

During the implementation period, 241 donors donated milk. In all, 541 neonates received PDHM as a bridge when MOM was insufficient or temporarily unavailable. Most beneficiaries were preterm infants admitted to our NICU. Bacterial growth was detected in 58/591 (9.8%) processed samples on microbiological culture. Heavy microbial growth in PDHM samples in September (20/47, 42.6%) and October (13/46, 28.3%) affected team motivation and disrupted workflow; however, corrective actions restored normal operations (Table 5; Figure 5).

**Table 5:** Month-wise performance of the CLMC. The total donated volume (140,088 mL) includes contributions from all 241 registered donors. Pasteurisation and microbiological testing became fully operational on 17 July 2024; therefore, the counts for July 2024 reflect the shorter operational period.

Month	Number of donors	Volume of milk donated (mL)	Number of neonatal beneficiaries	Culture status	
				Sterile	Unsterile
July 2024	6	410	29	4	0
August 2024	11	8,695	48	47	6
September 2024	15	6,558	55	27	20
October 2024	18	5,653	39	33	13
November 2024	18	9,900	47	28	5
December 2024	20	6,320	39	45	0
January 2025	18	10,287	53	54	3
February 2025	18	10,260	39	46	1
March 2025	27	9,185	37	43	2
April 2025	25	11,135	39	54	4
May 2025	16	6,581	24	35	0
June 2025	19	25,095	33	43	3
July 2025	30	30,009	59	74	1
Total	241	140,088	541	533	58 (9.8%)

**Figure 5:**
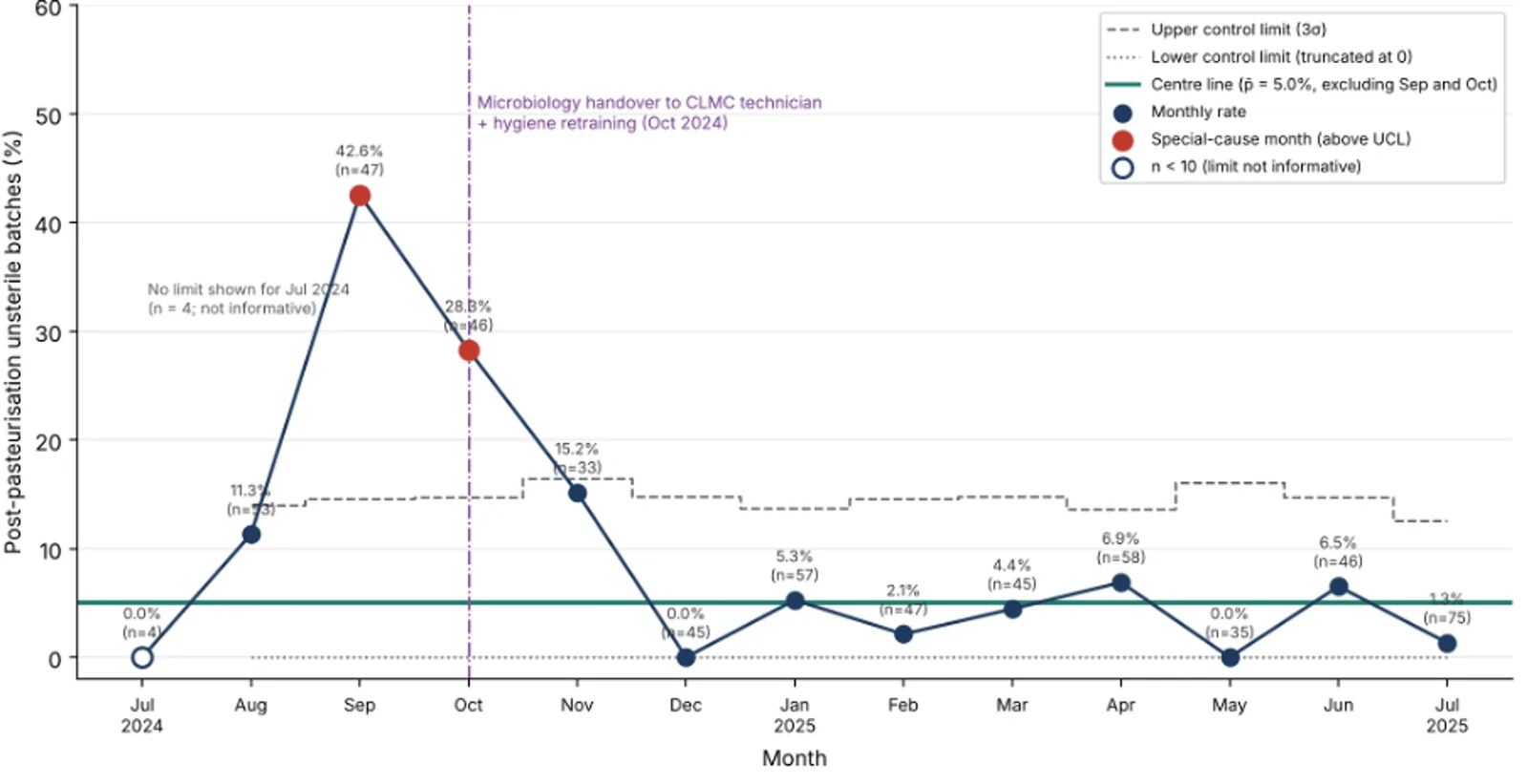
Statistical process control (SPC p-chart) of the monthly post-pasteurisation contamination rate, with the centre line (5.0%, excluding September and October) and 3-sigma control limits. Monthly post-pasteurisation contamination rate, July 2024-July 2025. Limits were calculated using non-special-cause months (*n* = 591 batches; 58 unsterile).

## Discussion

This retrospective implementation study describes the operationalisation of a CLMC in a high-volume public-sector tertiary NICU in North India and documents improvements in EBF and structured breastfeeding counselling coverage. The proportion of neonates exclusively breastfed at discharge nearly doubled, and a functional donor human milk pathway was established and used.

The gains are best understood as the combined effect of several operational components rather than any single input. Early expression, particularly hand expression, enabled mothers of sick and preterm neonates to build and maintain their milk supply during the first few days, when separation from the infant is unavoidable.

Structured support within a dedicated service also helps the unit adhere consistently to the revised Baby-friendly Hospital Initiative (BFHI) [9]. BFHI practices, structured post-partum lactation counselling and NICU feeding protocols are central to the CLMC's function and align with breastfeeding-centred care [12]. In a systematic review and meta-analysis by Sinha et al., interventions to improve feeding outcomes increased EBF rates by 44%. Among the interventions, counselling had the greatest effect on EBF, whether provided in hospital (RR 1.66, 95% CI 1.43-1.92) or at home (RR 1.58, 95% CI 1.39-1.80) [13]. Another systematic review of interventions in LMICs found that programmes spanning the antenatal to postnatal period and delivered through multiple contacts also had larger effects on EBF at 6 months (RR = 2.19) [14]. The reductions in formula use and uptake of PDHM that we observed are consistent with reports from public-sector facilities in India [15]. We did not report neonatal outcomes such as necrotising enterocolitis (NEC) or sepsis.

The median volume donated per donor was 185 mL (IQR 83.75-391.25). Other Indian studies reported mean volumes per donor of 268 ± 386 mL and 77.62 ± 51.26 mL, respectively [16,17]. Because of extreme values in our dataset, we reported the volume per donor as a median; therefore, a meaningful comparison with these studies is not possible. The lower volumes compared with those reported in developed nations reflect the structural realities of LMICs and highlight the need for sustained donor recruitment and effective lactation support.

Globally, the mean volume donated per donor is much higher (3L/donor-40L/donor) [18,19].

Programmes are better structured in developed nations, where donors are often recruited through awareness campaigns, are motivated to donate and frequently continue donating over the long term [20-22]. The post-pasteurisation contamination rate in our study was high compared with rates reported in other studies [16,17]. Simple handwashing techniques and proper cleaning and sterilisation of equipment used in milk collection, processing and storage help reduce contamination by hospital microbiota [23]. Educational interventions using video messages and awareness activities also reduced contamination, from 62/244 (25.4%) to 20/244 (8.2%), in the study by Karimi et al. [24].

Mean maternal age was 27.06 ± 3.39 years in the term group and 27.26 ± 4.42 years in the preterm group. Other Indian studies have also reported that most donors were in the 21-30-year age group [16,17]. Data from developed countries, by contrast, show older donors, with most mothers aged 30-39 years [25]. Early marriage in LMICs means that most pregnancies, and therefore most donors, occur among younger women. Studies from developing countries have reported a higher proportion of primiparous donors, but we did not observe this in our study [6,26]. In developed countries, mothers are often screened for health conditions, are educated and tend to lead healthy lifestyles. In LMICs, the higher burden of malnutrition and pregnancy-related health problems increases the risk of premature and operative deliveries, while structured support during the hospital stay is associated with higher donation rates [16,17]. We found that mothers of preterm neonates donated a larger share of the total milk volume. In a study of donor profiles in an LMIC, most donor mothers had delivered preterm infants (1,018/1,553 (65.5%)); NICU stays were longer, and mothers of NICU-admitted infants donated significantly more milk than mothers in the postnatal ward (*P* = 0.0001) [16].

System-level insights

Although the CLMC was structured, its functioning depended on continuous reinforcement. Donor recruitment, adherence to milk expression and documentation all required ongoing counsellor engagement, and the gains would probably regress without sustained staffing and supervision. Mother-infant separation was the barrier most consistently documented in operational records, although we did not formally assess its quantitative effect on lactation outcomes. This suggests that scaling up a CLMC in similar settings should include explicit provision for counselling time, hands-on expression support, coordination platforms and continuous mentorship, rather than focusing solely on equipment or physical infrastructure. The model could be replicated within the government healthcare system to address the initial operationalisation challenges of establishing a CLMC.

Strengths and limitations

Strengths of this study include its real-world tertiary public-sector setting, the relatively long implementation period of over a year, the use of both process (counselling coverage) and outcome (feeding practice at discharge) indicators, and the documentation of operational adaptations that are rarely reported in implementation literature from this context.

The main limitations are the retrospective design and the lack of a parallel control group. Baseline data were drawn from routine pre-CLMC clinical records, which captured counselling and feeding practices less completely than the prospective registers maintained during implementation. This data asymmetry introduces a risk of measurement bias and may overestimate the effect size. The before-after comparisons are descriptive, and inferential testing was not applied. We could not extract neonatal clinical outcomes from baseline records with sufficient completeness for comparison. The proposed mechanisms of change were inferred from routine documentation, rather than from formal qualitative methods (e.g., interviews or focus groups), and should be interpreted as hypotheses. Microbial identification was not performed because it was unavailable at our centre. The segmented interrupted time-series analysis was based on only three monthly baseline data points, limiting the precision of the estimated pre-intervention slope.

## Conclusions

Operationalising a CLMC in a public-sector tertiary NICU was associated with improvements in structured breastfeeding counselling coverage and exclusive breastfeeding at discharge. Our experience suggests that counselling, early bedside expression support, KMC integration, daily coordination and responsive problem-solving may have contributed to the observed improvements. However, the study design precludes valid causal inference. These findings may inform consideration of CLMC operationalisation in similar tertiary settings in LMICs, with attention to staffing, coordination and continuous mentorship.

## Appendices

Appendix A 

**Table 6:** StaRI (Standards for Reporting Implementation Studies): completed checklist Study: Operationalising a Comprehensive Lactation Management Centre in a Tertiary NICU in North India: a retrospective implementation study with before-after comparison of Breastfeeding and Counselling Coverage. Dual strands: the implementation strategy = how the CLMC was embedded (LIFT-UP facilitation, rounds, review meetings, coordination, audit); the intervention = the CLMC service itself (counselling, expression support, PDHM pathway). The paper largely treats these as one.

Item	Checklist item	Implementation strategy: how addressed	Intervention: how addressed	Where reported (section)
Title and abstract				
1	Title Identification as an implementation study; methodology in title/keywords.	The title identifies the study as a retrospective implementation study with a before-after comparison of Breastfeeding and Counselling Coverage.		Title; Keywords.
2	Abstract Identify as implementation study; describe strategy tested, evidence-based intervention, key implementation & health outcomes.	The abstract presents the CLMC as the intervention being operationalised and reports both operational indicators (structured counselling coverage, donor numbers, PDHM recipients and peak monthly donation volume) and exclusive breastmilk feeding at discharge		Abstract.
Introduction				
3	Introduction The problem/deficiency the intervention aims to address.	NICU feeding practices compromised by limited lactation support and mother-infant separation; EBF and early human-milk feeding remain suboptimal in LMIC NICUs despite strong evidence; CLMC implementation in high-volume public tertiary centres is uneven.		Introduction.
4	Rationale Scientific background & rationale (theory/framework, expected mechanism, pilot work).	The rationale is grounded in the 2017 National Guidelines on Lactation Management Centres and the recognised gap between policy and consistent ground-level functioning (staffing, competing clinical priorities, infrastructure constraints and the need for sustained behavioural support). A pragmatic, facility-based system-strengthening approach was adopted, supported by the LIFT-UP programme through training, supportive supervision, mentorship and coordination.	MOM is the recommended first feed, which reduces NEC and late-onset sepsis and improves neurodevelopment; PDHM is next-best when MOM is insufficient; counselling has strong evidence for improving EBF	Introduction; Discussion.
5	Aims and objectives Aims, differentiating implementation objectives from intervention objectives.	Three objectives are stated: to describe how the CLMC was operationalised and adapted (the implementation objective); to evaluate the effect on Breastfeeding and Counselling Coverage through a before-after comparison; and to characterise donor-milk collection, utilisation, microbiological quality and the donor profile.		Introduction (final paragraph).
Methods: description				
6	Design Design and key features of the evaluation (cross-reference methodology standards); protocol changes with reasons.	Retrospective implementation study with a before-after comparison; baseline April to June 2024 versus implementation July 2024 to July 2025. Baseline outcomes were drawn from routine clinical records and implementation data from prospectively maintained CLMC registers, and operational adaptations were documented iteratively through the service registers and review meetings.		Study Design and Setting; Statistical Analysis.
7	Context The context in which the intervention was implemented (barriers/facilitators relevant elsewhere).	High-volume public-sector tertiary teaching hospital serving eastern Uttar Pradesh and adjoining Bihar; 4200–4500 deliveries/year; overcrowded NICU; resource constraints; NHM-UP funding. Barriers: mother-infant separation, perceived low milk supply, limited dedicated staff, competing clinical priorities. Facilitator: national policy endorsement.		Introduction; Study Design and Setting; Discussion.
8	Targeted ‘sites’ Characteristics of targeted site(s) and eligibility; population targeted by the intervention.	40-bedded Level-III NICU plus a dedicated CLMC area and postnatal wards; personnel include counsellors, staff nurses, NICU clinicians, CLMC manager, milk-bank/CLMC technician, microbiology and hygiene staff.	Neonates admitted to the NICU; postpartum mothers who were screened and voluntarily donated after satisfactorily feeding their own baby. Exclusions: critically sick mothers, mothers ineligible for donation, and incomplete records.	Study Design and Setting (setting; inclusion/exclusion).
9	Description A description of the implementation strategy; a description of the intervention.	LIFT-UP facilitatory programme (training, supportive supervision, mentorship); daily multidisciplinary rounds with per-neonate feeding-plan review; weekly/monthly operational review meetings; WhatsApp-based coordination; structured event log/audit; dedicated space and staffing; iterative adaptation. Cross-ref: intervention detail itemised in the completed TIDieR checklist.	CLMC service components: structured breastfeeding counselling; bedside/hand milk-expression support; PDHM pathway (screening, pooling <100 mL, Holder pasteurisation, microbiological checks, cold-chain, prescription-based issue); behaviour-change support; KMC integration.	Description of the Intervention; Operational Review Mechanism; Results.
10	Sub-groups Any sub-groups recruited for additional research tasks / nested studies.	Donor mothers form a nested descriptive sub-study: 212 of 241 analysed (117 term, 95 preterm); 29 excluded for incomplete records. No other nested studies.		Results (donor profile); Figure 3 (flow).
Methods: evaluation				
11	Outcomes Pre-specified primary and other outcomes and how assessed; pre-determined targets.	The primary outcomes were the proportion of mothers receiving structured breastfeeding counselling and, at NICU discharge, the proportions exclusively breastfed and receiving formula. Operational indicators included unique donors, neonates receiving PDHM, peak monthly donation volume and post-pasteurisation microbiological status. Outcomes were assessed against the pre-CLMC baseline period.	EBF at NICU discharge defined	Data Collection (outcomes); Before-After Comparison.
12	Process evaluation Process-evaluation objectives/outcomes related to the strategy’s mechanism.	A formal, pre-specified process evaluation was not undertaken as part of this operational report. The proposed mechanisms of change (early initiation of expression, hand expression, demonstration-based teaching, KMC integration and daily coordination) were inferred from routine operational documentation and are presented as hypotheses.		Results (Establishment; Implementation Experience); Discussion; Limitations.
13	Economic evaluation Methods for resource use, costs, economic outcomes and analysis.	Not reported. No costing/resource-use analysis of the implementation strategy.	Not reported. No cost analysis of the intervention (service is NHM-UP funded).	Not applicable.
14	Sample size Rationale for sample sizes (calculations, budgetary/practical constraints, saturation).	Descriptive design: no formal sample-size calculation. Baseline audit: 353 admissions (counselling coverage) , breastfeeding assessed among neonates who were more sicker in initial days ,later were shifted to mother newborn care unit (MNCU) and had a documented discharge feeding mode (baseline n=56; implementation n=534). Donor descriptive analysis n=212.		Study Design (baseline); Results.
15	Analysis Methods of analysis, with reasons.	Breastfeeding and Counselling Coverage proportions were compared descriptively as absolute difference and as risk ratios. Risk ratios don’t reflect causation (Table 2). The monthly exclusive-breastfeeding trend was assessed by a segmented (interrupted time-series) regression across three baseline and 13 implementation months (immediate level change +36 percentage points, 95% CI 24.6 to 47.2, p<0.001; binomial segmented regression with quasi-binomial standard errors estimated a level change of OR 5.38 (95% CI 1.61–17.9). Donor characteristics compared with chi-square or Fisher's exact test and the independent-samples t-test (Table 3). Donation volume, dichotomised at sample median >185 mL versus <_ 185 mL, was analysed by multivariable binary logistic regression (Table 4). JASP v0.96.0 was used for donor characteristics and multivariable logistic regression ,and remaining part in Stata v19.5 ; tests two-tailed, p<0.05.		Statistical Analysis.
16	Sub-group analyses Any a priori sub-group analyses and sub-groups in nested tasks.	Donor mothers were compared by gestational category (term versus preterm delivery; Table 3) as part of the planned donor-profile analysis.		Statistical Analysis; Results (donor profile).
Results				
17	Characteristics Proportion recruited and characteristics of the recipient population.	Sites/personnel described narratively; conventional “proportion recruited” not applicable to the strategy.	Donor mothers were characterised, predominantly caesarean delivery; comparable parity, occupation and residence between groups; 212 of 241 donors analysed). Neonatal recipients were predominantly preterm or low-birth-weight infants. Breastfeeding and Counselling Coverage are reported in Table 2.	Results (donor profile); Figure 2
18	Outcomes Primary and other outcomes of the strategy / intervention.	Structured counselling coverage rose 25%→88%; 241 donors; 541 neonates received PDHM; peak monthly donation 30,009 mL; unsterile PDHM 58/591 (9.8%) overall.	EBF at discharge 45%→82 % (register cohort; RR 1.83)	Results (Quantitative Outcomes).
19	Process outcomes Process data mapped to the mechanism by which the strategy works.	Process observations were recorded narratively: early initiation of expression was associated with higher documented milk output; hand expression predominated for very preterm or sick neonates; the CLMC functioned as a problem-solving referral hub; and the most persistent barriers were mother-infant separation and perceived low milk supply.		Results (Implementation Experience; Challenges & Adaptations).
20	Economic evaluation Resource use, costs, economic outcomes and analysis.	Not reported.	Not reported.	Not applicable.
21	Sub-group analyses Representativeness and outcomes of sub-groups.	Among donors analysed (212 of 241; 29 excluded for incomplete records, Figure 3), mothers of preterm infants contributed a larger share of the donated volume (69.6% versus 30.3%) and had lower rates of skin-to-skin contact (26.3% versus 68.4%; p=0.006) and early breastfeeding initiation (18.9% versus 56.4%; p<0.001), with higher NICU admission and maternal risk-factor rates.		Results (donor profile); Table 3.
22	Fidelity / adaptation Fidelity to the strategy & adaptation to context; fidelity to core intervention components.	Fidelity and adaptation were monitored through weekly and monthly review meetings and, from December 2024, a structured event log. Documented adaptations included transfer of microbiology processes to the CLMC technician (October 2024), retraining of hygiene staff, revised counselling content, strengthened night-shift expression support and WhatsApp-based coordination. Fidelity was tracked pragmatically through service indicators and per-neonate feeding-plan documentation.	Core components largely delivered: counselling coverage 88%; feeding plan documented for every neonate; 2–3 hourly expression as SOP. Weakness: donor recruitment required continuous outreach and did not become self-sustaining.	Operational Review Mechanism; Results (Challenges & Adaptations).
23	Contextual changes Contextual changes that may have affected outcomes.	Reassignment of microbiology responsibility to the CLMC technician; hygiene retraining after audit; establishment of the event log; a seasonal post-pasteurisation contamination spike (Sept 42.6%, Oct 28.3%) that disrupted workflow and team morale before corrective action. No major external policy change reported during the study.		Results (Challenges & Adaptations).
24	Harms All important harms or unintended effects.	Neonatal outcomes were not captured. As a safety measure, unsterile pasteurised donor-milk batches (58 of 591; 9.8%) were discarded, with a transient contamination rise in September and October 2024, attributed to handling and hygiene lapses, that was subsequently corrected. Neonatal clinical outcomes such as necrotising enterocolitis and late-onset sepsis were not captured in this operational dataset, as noted in the Limitations.		Results; Strengths and Limitations.
Discussion				
25	Structured discussion Summary, strengths & limitations, comparisons, conclusions and implications.	Summary: EBF at discharge nearly doubled, functional PDHM pathway established. Strengths: real-world public-sector setting, 13 month period, process + outcome indicators, documented adaptations. Limitations: retrospective, no control, baseline-post measurement asymmetry (bias risk), no inferential testing of feeding outcomes, no neonatal outcomes, inferred mechanisms, no organism identification, sustainability not assessed. Comparisons drawn with Indian and other LMIC/HIC studies.		Discussion; Strengths and Limitations; Conclusion.
26	Implications Implications for policy/practice/research: scalability (strategy) and sustainability (intervention).	Supports policy-driven scale-up of CLMCs in similar LMIC tertiary settings; scale-up should explicitly budget for counselling time, hands-on expression support, coordination platforms and continuous mentorship rather than equipment/infrastructure alone.	Gains depend on continuous reinforcement (sustained staffing and supervision) and would likely regress without it; sustainability beyond the study period was not assessed.	Discussion (System-Level Insights); Conclusion; Limitations.
General				
27	Statements Regulatory/ethical approvals, use of routine data, registration/protocol, funding, conflicts.	Ethical approval: IMS-BHU (Ref IMS/IEC/FEBRUARY/2026/319, dated 12.03.2026). Data: routine records used, de-identified before analysis. Funding: NHM-UP (no separate grant). Conflicts: none. LLM use disclosed. Gaps: CTRI registration not done.		Methods (ethics); Declarations; Acknowledgements.

Appendix B

**Table 7:** The TIDieR (Template for Intervention Description and Replication) Checklist

Item #	TIDieR item	How addressed in this study	Where located (manuscript section) / Other
1	BRIEF NAME Provide the name or a phrase that describes the intervention.	Comprehensive Lactation Management Centre (CLMC) operationalised within a Level-III NICU, integrating structured breastfeeding counselling, bedside milk-expression support and a pasteurised donor human milk (PDHM) pathway within routine NICU workflow (distinct from a stand-alone donor milk bank). Implementation was supported by the LIFT-UP system-strengthening facilitatory programme.	Title; Introduction (final paragraph); Description of the Intervention.
2	WHY Describe any rationale, theory, or goal of the elements essential to the intervention.	Rationale: Mother’s own milk (MOM) is the recommended first feed for sick/preterm neonates; EBF and early human-milk feeding in LMIC NICUs remain suboptimal despite evidence of reduced NEC, late-onset sepsis and improved neurodevelopment; PDHM is the next-best option when MOM is insufficient or unavailable. The MoHFW National Guidelines (2017) recommend CLMCs integrating breastfeeding promotion, expression support and donor-milk banking on a single platform. Goal: Improve feeding outcomes (EBF at discharge ) and establish a functional PDHM pathway. Implicit theory of change: Process-based strengthening (early initiation of expression, hands-on demonstration, KMC integration, daily coordination and responsive problem-solving) rather than any single technological/infrastructural input.	Introduction; Discussion (mechanisms; System-Level Insights). Other: MoHFW National Guidelines 2017 (ref 5; nhm.gov.in).
3	WHAT: Materials Physical or informational materials used in the intervention (incl. those given to participants or used in delivery/provider training); where they can be accessed.	Dedicated CLMC expression space within the existing NICU; breast pumps with suction kits and hand-expression support; a Holder pasteurisation unit; cold-chain storage; donor-screening protocols following the 2017 National Guidelines; and post-pasteurisation microbiological culture. Documentation tools comprised the CLMC service register, milk-bank log, postnatal round registers, feeding records and, from December 2024, a structured event log, together with iteratively revised counselling content.	Description of the Intervention; Results (Establishment & Initial Operating Model). Information material for counselling included flow charts, pictorial demonstration and expression technique counselling on breast model. Not located in manuscript. Other: Donor screening per MoHFW 2017.
4	WHAT: Procedures Describe each procedure, activity and/or process, including any enabling or support activities.	(a) Structured delivery-room and postnatal breastfeeding counselling; (b) bedside/dedicated-room expression support with demonstration-based hand expression on 2–3 hourly cycles; (c) PDHM pathway: donor screening, hygienic collection, pooling when a single donor’s volume <100 mL, Holder pasteurisation, post-pasteurisation microbiological checks, cold-chain storage, and prescription-based PDHM issue when MOM insufficient/unavailable; (d) daily multidisciplinary rounds with per-neonate feeding-plan review (MOM availability, volume, mode); (e) WhatsApp-based real-time coordination; (f) structured behaviour-change support (perceived low supply, sustaining expression during separation, KMC integration); (g) weekly/monthly operational review meetings.	Description of the Intervention; Operational Review Mechanism; Results.
5	WHO PROVIDED For each provider category, describe expertise, background and any specific training given.	Lactation counsellors and dedicated counselling staff; staff nurses providing bedside expression demonstration; NICU clinicians; the CLMC manager; the milk-bank and CLMC technician, who took on microbiology-related processes from October 2024; the microbiology laboratory team; and hygiene staff, retrained after audit. Training and support were delivered through the LIFT-UP facilitatory programme, comprising structured training, supportive supervision and mentorship. All staff posted in NICU, SNCU, and Labour room were trained in workshops.	Description of the Intervention; Results (Challenges & Adaptations); Acknowledgements.
6	HOW Describe the mode(s) of delivery (e.g. face-to-face or other) and whether provided individually or in a group.	Delivery was predominantly face-to-face and individual, at the mother's bedside, combining counselling with hands-on expression demonstration. Staff-side coordination was handled digitally through a WhatsApp group linking clinicians, nurses and the CLMC manager, particularly for evening and night-time queries.	Description of the Intervention.
7	WHERE Type(s) of location(s) where the intervention occurred, including necessary infrastructure/relevant features.	40-bedded Level-III NICU of a public-sector tertiary teaching hospital, with a dedicated CLMC area and adjoining postnatal wards. Catchment: eastern Uttar Pradesh and adjoining districts of Bihar; ~4200–4500 deliveries/year; high proportion of preterm/LBW/referred-in sick neonates. Infrastructure: Dedicated expression space, pasteurisation unit, cold-chain storage, on-site microbiology laboratory.	Materials & Methodology (Study Design and Setting).
8	WHEN & HOW MUCH Number of times delivered and over what period: sessions, schedule, duration, intensity or dose.	Implementation period July 2024 to July 2025 (about 13 months); baseline April to June 2024. Dose: structured counselling = at least one session with hands-on expression demonstration; expression initiated early (within the first hour and first day of admission) and repeated during daily multidisciplinary rounds; weekly/monthly review meetings. Counselling was recorded as overall coverage and contacts, as a mother could receive multiple sessions: 2,155 CLMC counselling contacts and 6,864 in-ward counselling sessions were delivered over the period.	Study Design; Description of the Intervention; Results.
9	TAILORING If planned to be personalised, titrated or adapted, describe what, why, when and how.	PDHM individualised as a bridge only when MOM insufficient/unavailable; hand expression preferred for mothers of very preterm/sick neonates in early postpartum days; counselling tailored to individual barriers (perceived low supply, prolonged mother-infant separation); donor milk pooled when a single donor’s volume was <100 mL to meet minimum batch volume.	Description of the Intervention; Results (Implementation Experience).
10	MODIFICATIONS If modified during the study, describe the changes (what, why, when, how).	Oct 2024: microbiology-related processes transferred to the CLMC technician to address high post-pasteurisation contamination (Sept 42.6%, Oct 28.3% of batches unsterile); hygiene staff retrained after an audit found dust accumulation and inadequate cleaning of the pump suction-kit valve diaphragm. Dec 2024: structured event log introduced to audit data-entry discrepancies and PDHM-handling errors (especially night hours). WhatsApp coordination added for after-hours queries; counselling content revised to address perceived insufficient milk; night-shift expression support strengthened.	Results (Challenges & Adaptations); Operational Review Mechanism.
11	HOW WELL: Planned If adherence/fidelity was assessed, describe how and by whom, and any strategies used to maintain/improve it.	Implementation was monitored through weekly and monthly review meetings (service volumes, donor availability and bottlenecks) and, from December 2024, a structured event log. Fidelity was tracked through the counselling-coverage indicator and per-neonate feeding-plan documentation, with supportive supervision and mentorship through LIFT-UP used to sustain it. Monitoring was pragmatic and register-based rather than through a formal quality-improvement methodology.	Operational Review Mechanism; Data Collection; Results.
12	HOW WELL: Actual If adherence/fidelity was assessed, describe the extent to which the intervention was delivered as planned.	Structured counselling coverage (unique mothers) rose 25%→88%, delivered as 2,155 CLMC counselling contacts and 6,864 in-ward sessions (authors caution the coverage rise partly reflects improved documentation relative to baseline records); feeding plans documented for every admitted neonate; 541 neonates received PDHM, 241 donors registered, peak monthly donation 30,009 mL; exclusive breastfeeding at discharge rose from 45% to 82% Delivery gaps: Donor recruitment required sustained counsellor outreach and did not become self-sustaining; contamination episodes transiently disrupted workflow before corrective action restored operations.	Results (Quantitative Outcomes; Challenges & Adaptations).
